# Keeping mtDNA in Shape between Generations

**DOI:** 10.1371/journal.pgen.1004670

**Published:** 2014-10-09

**Authors:** James B. Stewart, Nils-Göran Larsson

**Affiliations:** 1 Department of Mitochondrial Biology, Max Planck Institute for Biology of Ageing, Cologne, Germany; 2 Department of Laboratory Medicine, Karolinska Institutet, Stockholm, Sweden; University of Miami, United States of America

## Abstract

Since the unexpected discovery that mitochondria contain their own distinct DNA molecules, studies of the mitochondrial DNA (mtDNA) have yielded many surprises. In animals, transmission of the mtDNA genome is explicitly non-Mendelian, with a very high number of genome copies being inherited from the mother after a drastic bottleneck. Recent work has begun to uncover the molecular details of this unusual mode of transmission. Many surprising variations in animal mitochondrial biology are known; however, a series of recent studies have identified a core of evolutionarily conserved mechanisms relating to mtDNA inheritance, e.g., mtDNA bottlenecks during germ cell development, selection against specific mtDNA mutation types during maternal transmission, and targeted destruction of sperm mitochondria. In this review, we outline recent literature on the transmission of mtDNA in animals and highlight the implications for human health and ageing.

## Introduction

Evidence continues to accumulate that a fusion event between an α-proteobacteria and an archaebacteria is the defining event in the evolution of the eukaryotic cell. Though it has been speculated that other endosymbiotic events may have been involved in the organellar structure of the eukaryotic cell, genomic evidence to date supports the presence of only α-proteobacterial and archaebacterial genomes at the beginning of this fusion [1]. Indeed, all eukaryotes sampled to date contain an organelle of mitochondrial origin and have genes of the mitochondrial ancestor within their nuclear genomes [2]. As with most endosymbiotic associations, the mitochondrial ancestral genome quickly began to lose genes that were no longer needed within their new host cell environment. Though we often shorthand the reassignment of mitochondrial genes as “being transferred to the nucleus”, this is only literally true for a minority of genes. An early study in yeast showed that only about 10% of the nuclear genes encoding mitochondrial proteins are clearly derived from the ancestral α-proteobacterial genome [3]. In mice and humans, only ∼35% of the gene products targeted to the mitochondria have good matches to the proteome of *Rickettsia* [4], whereas the remaining mitochondrial-targeted proteins mostly are derived from nuclear genes of nonmitochondrial origin that have displaced or complemented functions lost as the mitochondrial genome contracted. Other mitochondrial proteins are clearly the result of unusual horizontal gene transfer events, such as the replacement of the genes involved in mtDNA replication and transcription by T-odd bacteriophage enzymes [5].

Throughout eukaryotic life, very different evolutionary states of the mitochondria have arisen, and nearly all generalities to describe the mitochondria have been broken in various taxa [6]. All aerobic mitochondria appear to have retained a small mtDNA genome [7]–[9], encoding at least the *cox1*, *cox3*, and *cytb* genes as well as remnants of the rRNA genes [10], [11]. Anaerobic mitochondria differ in their requirements of an independent mtDNA molecule. There are anaerobic mitochondria and hydrogen-producing mitochondria, which have altered function of their respiratory chain, but still maintain mtDNA. However, the loss of mtDNA in hydrogenosomes or mitosomes is also well documented [2]. The path to an mtDNA-free organelle appears to have occurred independently in multiple lineages, and preliminary reports suggest that even a recently described anaerobic animal has an organelle, which visually resembles a hydrogenosome [12], [13], although detailed biochemical and genetic characterization of this organelle still remains to be done.

We will constrain this review to a discussion of mitochondrial genetics in animals, with special focus on principles for inheritance and purifying selection of mtDNA in the mammalian maternal germline. Most animal mitochondrial genomes conform to a specific genome composition, but even within animals, a surprising level of variation has recently been observed [14]. The genes encoded by the mtDNA are typically packed tightly together, with minimal noncoding DNA. One conspicuously large noncoding region goes by various names, including the control region, D-loop region, or large noncoding region. This region contains regulatory elements for transcription and replication of mtDNA [15]. The mammalian mtDNA encodes 13 protein components of the oxidative phosphorylation system (subunits of complexes I, III, IV, and V), highly reduced small and large rRNAs, and a minimal array of 22 tRNAs to decode the simplified animal mitochondrial genetic codes [16]. Occasionally, the observation of incomplete complements of tRNAs in some animal groups [17] have led to the hypothesis of mitochondrial tRNA import [18]. Furthermore, recent RNA sequencing has shown high levels of nuclear encoded tRNAs in RNA preparations from purified mitochondria [19]. However, the observation of cryptically encoded tRNAs in diverse animals groups [20]–[23] questions the true absence of a complete set of mtDNA-encoded tRNAs. Additionally, groups within the Porifera and Cnideria have lost all of the tRNA genes except one or two that are essential for maintaining their mitochondrial genetic codes [24], [25]. Presumably, after the origin of RNA import into the mitochondria in these animals, the mitochondrially-encoded tRNAs, which share their codons with imported nuclear-derived tRNAs, would have become functionally redundant and spared the evolutionary pressure to conserve their sequence. The elevated mutational load would therefore have led to loss of functional mtDNA genes encoding these redundant tRNAs. Our experience is that it is extremely challenging, if not impossible, to obtain mtDNA preparations free of nuclear DNA contamination, and it is therefore unlikely that mitochondrial RNA pools completely free of cytosolic contamination can be isolated [26]. Validation of the import hypothesis in animals will require the identification of the molecular pathway dedicated to the import of cytosolic tRNAs, as found in other eukaryotic groups that utilize cytosolic tRNAs for mitochondrial translation [27].

## Mitochondrial Transmission

**Figure 1:**
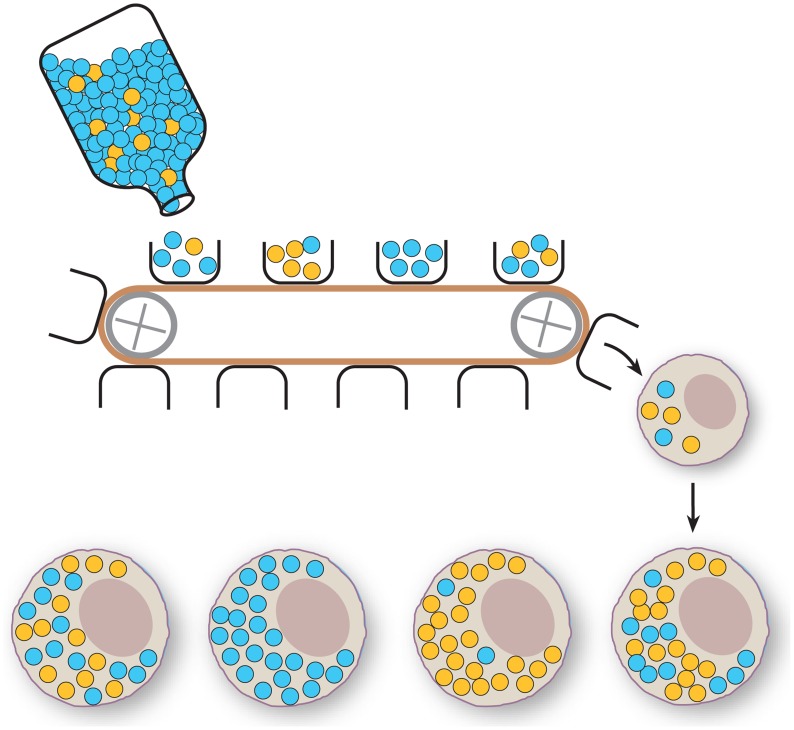
Schematic representation of the mitochondrial bottleneck. A large number of mtDNA molecules are present in the maternal mtDNA pool (bottle). The figure depicts two genotypes (blue and yellow circles). The generation of an oocyte involves subsampling of just a few mtDNA molecules from the maternal mtDNA pool (buckets on conveyor belt), which will be transferred to the developing oocyte and extensively replicated. The effect of this poorly understood bottleneck mechanism is that the proportion of the two genotypes can vary widely between oocytes.

The prokaryotic origins of the mitochondria also influence their genetics. In animals, most cells contain many copies of mtDNA, and the transmission can be better imagined as thousands of independent genomes being segregated by stochastic mechanisms and relaxed, uncoupled replication, which is in stark contrast to the structured meiosis and mitosis of eukaryotic nuclear genomes [28]. Despite the high copy number of mtDNA, it was noted very early that mtDNA of vertebrates can switch from one type to another in a very short period of time, even in a single generation [29]–[31]. To explain this very rapid shift, the concept of the mitochondrial bottleneck was developed (Figure 1). In the development of the germline, a large population of mtDNAs are subsampled to a relatively small number. This subselected population may differ markedly from the source population, allowing rare alleles to, by chance, suddenly come to dominate the mtDNA pool. With a sufficiently small sample, as observed in Holstein cows, single-generation shifts of mtDNA genotype are possible [30], [31]. Such a bottleneck phenomenon has since been detected widely in vertebrates [32]–[34] and even in *Drosophila* [35]–[37], though the size of this subsampling bottleneck appears to be an order of magnitude larger in flies than in vertebrates.

Usually, animal mtDNAs are inherited solely through the female oocyte. Upon fertilization, molecular mechanisms that identify and eliminate the sperm mitochondria have been described [38]. A similar phenomenon has been recently described in *Drosophila* [39] and *Caenorhabditis elegans* [40], [41], implying that it may be an ancestral feature to most animals. This targeted destruction of paternal mtDNA has clearly been lost in some species such as the *Mytilis* mussels, in which double uniparental inheritance of mtDNA occurs [42]. Like most molecular mechanisms, these systems can be circumvented. Interspecific crosses of mice lead to transmission of low levels of paternal mtDNA in early embryos [43]. However, the paternally transmitted mtDNA in these interspecific crosses is overrun by the maternal mtDNA during development to create offspring that only contain maternally transmitted mtDNA [43], [44]. There are some reports of recombination between the divergent maternal and paternal mtDNAs [45], [46], and recombined molecules appear to be present in the double uniparental mtDNA inheritance mussels as well, but at much lower rates than those resulting from homologous recombination during nuclear meiosis [47]. However, recombination appears to be conspicuously absent in many vertebrate groups. Mice that segregate neutral alleles appear to lack recombination in PCR-free assays [48], even when maintained in a constant heteroplasmic state for more than 50 generations [49]. However, mice heteroplasmic for one deleterious mtDNA type and a second mtDNA type from a distantly related species appear to have small patches of sequence consistent with recombination events [48]. It should be mentioned that the presence of rare recombinant mtDNA molecules does not necessarily necessitate the existence of a molecular machinery for homologous recombination. Instead, such recombinant molecules may be the product of copy choice recombination whereby the mitochondrial DNA polymerase starts replication on one mtDNA template and shifts to another template to complete replication. It remains possible that in deleterious backgrounds, these types of rare events may lead to a fitness advantage for the organism or organelle, leading to the fixation of these rare events, as seen in the interspecies hybrids [45], [46].

## Molecular Basis of the mtDNA Bottleneck

**Figure 2:**
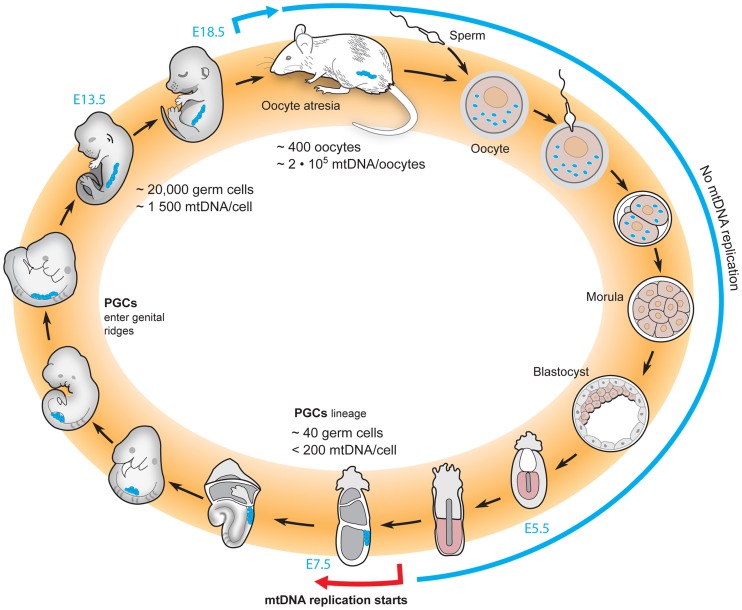
The life cycle of mtDNA within the female germline of mice. The total amount of mtDNA remains unchanged from the oocyte to the blastocyst stage, resulting in a dilution of the number of mtDNA molecules per cell with each cell division. Re-initiation of mtDNA replication does not occur until the postimplantation stage. At approximately E7.5, a physical bottleneck with a very low number of mtDNA molecules per cell has been reported, and thereafter replication reinitiates to increase the mtDNA content of the embryo. During the late stages of embryonic life and early postnatal life, oocytes are lost due to atresia.

In mice, the mitochondrial life cycle starts with ∼2×10^6^ copies of mtDNA in a single oocyte (Figure 2). After fertilization, designation of the primordial germ cells (PGCs) is delayed until after implantation of the embryo [50], [51]. During this stage, there is no gain in the amount of mtDNA in the embryo as a whole, effectively diluting the per-cell levels of mtDNA with each cell division [51]–[53]. Finally, mtDNA replication is re-initiated sometime between embryonic day (E)6 and E7.5, and the mtDNA copy number per cell begins to increase throughout embryogenesis [34], [51], [52]. There is further increase of mtDNA copy number during oocyte development in the postnatal period to reach the ∼2×10^6^ copies of mtDNA that are present in mature oocytes and passed on to the next generation [52], [54]. Though the oocyte development in *Drosophila* displays marked differences, a similar period without mtDNA replication has been observed, which could be expected to lead to an mtDNA bottleneck, albeit of a far less drastic nature than in mammals [36], [37].

There is an ongoing debate regarding when the mtDNA bottleneck occurs in the life cycle of mammalian mtDNA. Three different groups have used qRT-PCR based methods to assess the mtDNA copy number in the developing mouse germline, but have failed to reach a consensus [34], [53]–[57]. Two groups have concluded that a low number of mtDNAs is present at the early stages of the primordial germ cells [34], [54], and this low number approximates the bottleneck size estimated by measuring the variance of two neutral alleles segregating in mice [32]. One group argues that this event explains the mtDNA bottleneck [34], [57]. However, another group argues that the number of mtDNAs in early primordial germ cells is an order of magnitude higher than this low estimate and have reported that the bottleneck instead occurs via a subselection of mtDNAs that are preferentially replicated to fill the developing oocyte [53], [55]. Yet another group claims that the bottleneck occurs after birth, by a subpopulation of mtDNAs that supply the final replicative burst that populates the mature oocytes [54], [56]. Obviously, clarifying this point continues to be of great interest to the field. So far, only correlative data have addressed this important question and experiments will be needed to determine how changes in mtDNA copy number in the maternal germline influence the size of the bottleneck.

## Mutations in mtDNA

The most common types of mutations observed in the mtDNA are single nucleotide substitutions, single base insertions, single base deletions [58], and large deletions that result in smaller, circular mtDNA molecules [59], [60]. Other mutation types, such as multimers of sequences associated with replication elements [61], have been observed in the aging human brain [62], while deleted linear mtDNA molecules have only been seen in disease states or severely afflicted mouse models [63], [64]. The mtDNA copies within a cell may all be identical (homoplasmy) or contain two or more variants (heteroplasmy). It is common to refer to individual organisms as homoplasmic for an mtDNA type based on Sanger sequencing of amplified mtDNA pools. However, it is becoming clear that most individuals carry appreciable numbers of low-level mtDNA variants in their tissues [65]–[67]. One of us has recently reviewed the role of mtDNA mutations in ageing [68], and some recent additional insights will be further discussed here.

According to the classic mitochondrial theory of ageing, reactive oxygen species from the OXPHOS (Oxidative Phosphorylation) system will be mutagenic and lead to the accumulation of mtDNA mutations over time [69], [70]. However, recent studies have begun to cast doubt on this model and argue that normally occurring replication errors may be a more important source of mutations than unrepaired damage. Oxidative damage to DNA is predicted to create G>T transversions and C>T transitions. Unfortunately, only the G>T transversions are diagnostic when sequencing mtDNA because C>T transitions also appears to be a common replication error. It has been known for some time that there is a preponderance of transition mutations in human cell cultures, arguing that the observed mutational spectra are consistent with errors occurring during normal replication by mitochondrial DNA polymerase [71], [72]. The role of the oxidative adduct 8-oxo-deoxyguanosine in mtDNA mutagenesis was recently experimentally assessed in flies that were engineered to have a down-regulation of two base excision repair enzymes (OGG1/MUTYh) and mitochondrial superoxide dismutase [63]. This combined intervention is predicted to drastically increase 8-oxo-deoxyguanosine adducts and the formation of G>T transversion mutations, but, quite surprisingly, there was no increase in mtDNA mutations in these flies [73]. In fact, the observed mtDNA mutation pattern closely resembled the mitochondrial DNA polymerase error-based mutation pattern observed in wildtype flies [58]. Also, studies in ageing mice [26] and humans [62], [74] have failed to detect a transversion mutation bias as would be expected if elevated levels of oxidative damage would be the main source of mtDNA mutations.

A mutation will initially affect a single copy of mtDNA and each mutational event will therefore be exceptionally rare upon its generation, as most mammalian cells contain thousands of copies of mtDNA. However, the lack of coordination between the cell cycle and replication of mtDNA allows mitotic segregation of the mutated mtDNA copy, which thereby can undergo clonal expansion and over time overrun the mtDNA pool to cause physiological consequences. In fact, age-associated somatic mtDNA mutations, both point mutations and large deletions, are typically clonally expanded to create a mosaic pattern of respiratory chain deficient cells in ageing humans [75]–[77]. Characterization of point mutations in ageing humans have confirmed their apparently stochastic nature as sequencing shows no signature of purifying selection [78], [79]. A purely stochastic mutation profile is also observed in the coding regions of mtDNA isolated from the mtDNA mutator mouse, a substitution-prone model where the mitochondrial DNA polymerase has had its proofreading function disrupted [63], [80], [81]. Also, studies of patients with genetic disorders due to mtDNA mutations do not show a targeted reduction in the disease-causing deleterious alleles over time. These observations challenge the assertion that mitochondrial quality control mechanisms are able to target specific deleterious mtDNA molecules in somatic tissues.

However, there appears to be selection against somatic mtDNA mutations that affect sequences needed for mtDNA replication. Recent work has identified specific mutations, most commonly observed near the replication elements of the control region, which appear to undergo tissue-specific propagation in humans with age [82]. Similar mtDNA variants leading to replicative advantage have been observed in invertebrates [83], [84]. Negative selection against mutations in the origin of light strand replication, which controls initiation of lagging strand replication, has been observed in somatic tissues of mtDNA mutator mice [85]. Negative selection may also explain the decreased mtDNA mutation rate in the control region [63], [80].

If mtDNA mutations are present in the germline and pass through the oocyte, they rapidly expand to appreciable levels and end up in all tissues of the resulting organism [86]. Using the mtDNA mutator mice, we have recently shown that these germline–transmitted mtDNA mutations can severely affect the health of the resulting organism. By breeding successive generations of females that are heterozygous for the mtDNA mutator allele, we observed reduced litter sizes, impaired health, and shortened lifespan of the offspring. These effects in the offspring can be immediately reversed by the introduction of nonmutated mtDNA into the heterozygous mtDNA mutator females [86]. We have also been able to generate mice hemizygous for the mtDNA mutator allele that are functionally equivalent to classical mtDNA mutator mice, but lack maternally transmitted mutations from their mothers [86]. These mice are healthier than the standard mtDNA mutator mice and live, on average, ten weeks longer, thus showing that low levels of mtDNA mutations transmitted through the germline worsen the effects of somatic mutagenesis of mtDNA [86].

## Germline Purifying Selection

The high mutation rate of mtDNA, relative to the nuclear rate [58], [71], [72], and the absent or infrequent recombination of mtDNA are predicted to strongly increase the mutational burden of mtDNA [87]. The mitochondrial threshold effect allows disease alleles to accumulate without causing any disease phenotypes, but once the relative level of mutated mtDNA exceeds a critical threshold, devastating disease will occur [88]. Mothers of patients with mtDNA disease are often healthy despite having substantial levels of the disease-causing allele. However, the mtDNA bottleneck phenomenon allows the levels of a disease allele to be drastically elevated in a single generation, thus leading to mitochondrial disease in the offspring [89].

The famous geneticist Hermann Muller predicted that asexually transmitted genomes, such as mtDNA, would accumulate deleterious mutations over time, a phenomenon referred to as Muller's ratchet [90], [91]. Work during recent years has revealed strong mechanisms for purifying selection of mtDNA in the maternal germline that counteract a mutational meltdown. In mice, deleterious alleles of mtDNA that lead to amino acid substitutions are strongly selected against in the female germline. In experimental work, we introduced a random set of mtDNA mutations from mtDNA mutator mice into lineages of mice with a wildtype nuclear genome. Sequencing of mtDNA showed evidence of strong purifying selection because the levels of mtDNA mutations affecting the amino acid, altering first or second codon positions of protein coding genes, were much lower than the levels of mutations affecting the normally synonymous third codon position or tRNA genes [92]. The speed at which this selection occurred was very fast and was observable two generations after the introduction of mtDNA mutations from the mtDNA mutator mouse. These alleles are thus selected against when they are still very rare in the mtDNA pool and therefore can have no gross phenotypic effects on the organism itself. Independent support for purifying selection against mutations causing deleterious amino acid substitutions has been reported in mice with a deletion in *mt*-nd6, which was selectively eliminated in favor of a compensatory insertion mutation that corrected the reading frame [93].

The situation with mutations in protein coding genes is in stark contrast to mutations in tRNA genes of mtDNA. We studied a single-base deletion in the tRNA^Met^ gene, where mice could only be bred to harbor a maximum of 90% mutated mtDNA [84]. This mutation could accumulate to very high levels (∼100%) in the germline, but was selected against postfertilization in the embryonic period [94]. These experimental results showing quite different behaviors of mtDNA mutations affecting tRNA or protein coding genes likely provide an explanation for why tRNA mutations are frequent causes of mitochondrial disease in humans, whereas mutations in protein coding genes are rare. This discrepancy is especially striking when one takes into account that tRNA genes occupy 9% and protein coding genes 68% of the human mtDNA sequence.

Two recent studies in *Drosophila* have illustrated the deep conservation of mechanisms in the germline selecting against mtDNA mutations causing amino acid substitutions and have also added some interesting insights into how purifying selection can operate. These studies utilized a temperature sensitive mutation in the *mt-co1* gene, which has no phenotype at permissive temperatures, but is lethal when the flies are raised at 29°C [36], [37]. This temperature sensitivity allowed the generation of lines with very high relative levels of the mutant allele at permissive temperatures. By switching the line to high temperature, the allele was transmitted less efficiently and there was a selective lowering of its relative levels over a few generations. However, the allele was maintained at low levels for a very large number of generations, consistent with the predicted less drastic mtDNA bottleneck effect in the fruit fly [35]–[37].

The temperature sensitive nature of the mutation also allowed determination of when the allele was selected against in the germline. The selective event occurred with germline development in the flies and coincided with the re-initiation of mtDNA replication [37]. High levels of the deleterious allele led to the inability of the mitochondria to re-initiate mtDNA replication in the germline. These results led to the hypothesis that a decrease in the mitochondrial membrane potential will decrease import of nuclear gene products necessary for mtDNA replication. Such a mechanism could hamper the transmission of mtDNA mutations causing amino acid substitutions during the rapid mitochondrial biogenesis stage of oogenesis.

Now that the evolutionary conservation of this germline selection has been established, a more mechanistic study of the molecular or cell biological basis of the purifying selection is in order. A comparative genomics approach will be very useful in unraveling the mechanistic details of this phenomenon. Using the powerful genetics of *Drosophila*, it will be feasible to screen for the molecular pathways that could disrupt (or perhaps enhance) the selection against various deleterious alleles. Suitable mutant or RNAi stocks are available in *Drosophila*, including inducible mutants, such as the temperature sensitive mutants [36], [37]. For instance, the selection in *Drosophila* has already been determined to occur independently of the *PARKIN* pathway, as flies with *PARK* RNAi knockdown, or a null mutant of the *PARKIN* gene still showed germline selection against mutated mtDNA [36]. Results obtained in flies should then allow us to test for conserved mechanisms in mammals, such as the mouse, or eventually in humans.

## Concluding Remarks

The mtDNA is asexually transmitted in metazoans and should, in principle, be sensitive to the Muller ratchet phenomenon with irreversible mtDNA mutation accumulation between generations. However, studies in mammals have clarified that there are several mechanisms that can completely prevent or decrease the transmission of deleterious mtDNA mutations from one generation to the next: (I) The bottleneck phenomenon allows a mother with low levels of a pathogenic mtDNA mutation to produce offspring lacking mutated mtDNA, (II) there is a strong germline purifying selection against mtDNA mutations causing amino acid substitutions [82], (III) there is a strong purifying selection during embryogenesis against high levels of mtDNA mutations impairing tRNA function [84], and (IV) females with mtDNA mutations in the germline have a dose-dependent reduced capacity to reproduce [77]. Although recent progress has dramatically increased our knowledge of mechanisms that may reset or decrease the mtDNA mutation rate between generations, the involved molecular components are not at all understood. The challenge for the future will, therefore, be to understand how these mechanisms operate at a molecular level and how they have evolved.
